# Immune gene expression analysis indicates the potential of a self-amplifying Covid-19 mRNA vaccine

**DOI:** 10.1038/s41541-022-00573-y

**Published:** 2022-11-28

**Authors:** Eugenia Z. Ong, Jia Xin Yee, Justin S. G. Ooi, Ayesa Syenina, Ruklanthi de Alwis, Shiwei Chen, Jean X. Y. Sim, Shirin Kalimuddin, Yan Shan Leong, Yvonne F. Z. Chan, Rose Sekulovich, Brian M. Sullivan, Kelly Lindert, Sean B. Sullivan, Pad Chivukula, Steven G. Hughes, Jenny G. Low, Eng Eong Ooi, Kuan Rong Chan

**Affiliations:** 1 Program in Emerging Infectious Diseases, Duke-NUS Medical School, Singapore, Singapore; 2 Viral Research and Experimental Medicine Centre at SingHealth-Duke-NUS (ViREMiCS), SingHealth Duke-NUS Academic Medical Centre, Singapore, Singapore; 3 Department of Infectious Diseases, Singapore General Hospital, Singapore, Singapore; 4 Arcturus Therapeutics, Inc., San Diego, CA USA; 5 Saw Swee Hock School of Public Health, National University of Singapore, Singapore, Singapore

**Keywords:** Translational research, Predictive markers, RNA vaccines

## Abstract

Remarkable potency has been demonstrated for mRNA vaccines in reducing the global burden of the ongoing COVID-19 pandemic. An alternative form of the mRNA vaccine is the self-amplifying mRNA (sa-mRNA) vaccine, which encodes an alphavirus replicase that self-amplifies the full-length mRNA and SARS-CoV-2 spike (S) transgene. However, early-phase clinical trials of sa-mRNA COVID-19 vaccine candidates have questioned the potential of this platform to develop potent vaccines. We examined the immune gene response to a candidate sa-mRNA vaccine against COVID-19, ARCT-021, and compared our findings to the host response to other forms of vaccines. In blood samples from healthy volunteers that participated in a phase I/II clinical trial, greater induction of transcripts involved in Toll-like receptor (TLR) signalling, antigen presentation and complement activation at 1 day post-vaccination was associated with higher anti-S antibody titers. Conversely, transcripts involved in T-cell maturation at day 7 post-vaccination informed the magnitude of eventual S-specific T-cell responses. The transcriptomic signature for ARCT-021 vaccination strongly correlated with live viral vector vaccines, adjuvanted vaccines and BNT162b2 1 day post-vaccination. Moreover, the ARCT-021 signature correlated with day 7 YF17D live-attenuated vaccine transcriptomic responses. Altogether, our findings show that sa-mRNA vaccination induces innate immune responses that are associated with the development of adaptive immunity from other forms of vaccines, supporting further development of this vaccine platform for clinical application.

## Introduction

The coronavirus disease 2019 (COVID-19) pandemic has exerted a heavy toll on human lives, healthcare systems and socioeconomic well-being worldwide^1^. Fortunately, accelerated vaccine development and emergency use approval has now begun to change what was once a potentially life-threatening infectious disease into mostly asymptomatic infection and mild febrile illness^2^. Amongst these vaccines are the newly deployed mRNA vaccines, which have been consistently shown to be the most effective in preventing COVID-19^3^. An alternative form of the mRNA vaccine is the self-amplifying mRNA (sa-mRNA) vaccine. Such a vaccine is built using an alphavirus genomic backbone, with the gene encoding the antigen of interest, in this case the SARS-CoV-2 spike gene, spliced into the RNA backbone, replacing the alphavirus structural genes. Amplification of the spike gene would theoretically increase the abundance and duration of immunogen^4^ for better adaptive immune responses^5,6^ as compared to conventional mRNA vaccines. However, the COVID-19 sa-mRNA vaccine candidates have not shown robust immunogenicity in humans^7^. Defining if the sa-mRNA construct has the potential to produce potent vaccines would thus be important to shape future clinical development of this platform.

The innate immune response to vaccination shapes the development of adaptive immunity^8–10^. Indeed, induction of monocytes, neutrophils, Toll-like receptor signalling, inflammation and type I interferon response signatures were associated with antibody and T cell responses following BNT162b2 nucleoside-modified mRNA vaccination^11^. However, extensive activation of the innate immune response, especially type I interferons, may prematurely inhibit translation of sa-mRNA and possibly reduce the immunogenicity of sa-mRNA vaccines^12,13^. Our goal in this study was thus to define the innate immune response to sa-mRNA vaccination, and determine the extent to which the correlates of antibody and T cell responses to this vaccine compare with those elicited by other more studied forms of vaccines. We examined the human transcriptional response of immune genes in whole blood following vaccination with a candidate COVID-19 sa-mRNA vaccine, ARCT-021. ARCT-021 consists of a single-stranded mRNA bearing the genes encoding the replication complex of the Venezuelan equine encephalitis virus (VEEV) and the SARS-CoV-2 full-length spike (S) glycoprotein gene, and is packaged in a proprietary lipid nanoparticle^14^.

## Results

### Humoral and cell-mediated responses to ARCT-021 are weakly correlated

**Fig. 1:**
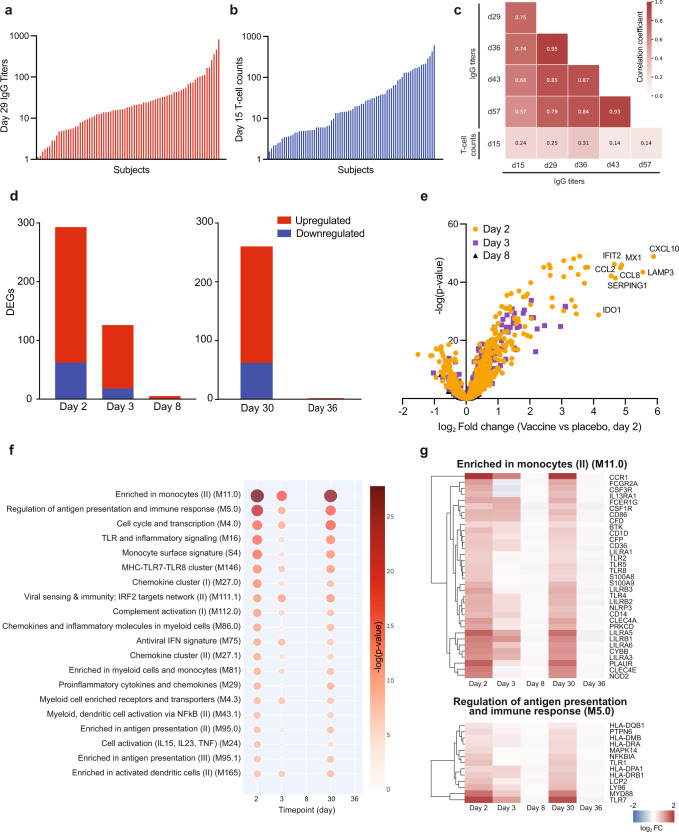
ARCT-021 triggers an early induction of transcripts related to innate immune responses and antigen presentation. **a** Day 29 IgG antibody titers and **b** Day 15 S-specific T cell responses in vaccinated individuals (*n* = 78), ranked from smallest to largest values. **c**. Correlation matrix showing the relationship of IgG titers (day 15, 29, 36, 43, 57) and day 15 S-specific T cell responses after ARCT-21 vaccination. The colour intensity is proportional to the correlation coefficient. **d**. Differentially expressed genes (DEGs) detected in all subjects receiving ARCT-021 (*n* = 78), as compared to the placebo group (*n* = 28) for day 2, 3, 8, 30 and 36. DEGs were identified based on fold-change >1.3 and false discovery rate-adjusted *p* value < 0.05 (Benjamini-Hochberg step-up procedure). Red bars indicate number of upregulated DEGs and blue bars indicate number of downregulated DEGs. **e** Volcano plot displaying genes that were most differentially expressed at day 2, 3 and 8 after vaccination. The most differentially regulated genes are annotated on the volcano plot. Volcano plot for day 30 and 36 are shown in Supplementary Fig. 1c. **f** Top 20 Blood Transcription Modules (BTMs) that are positively enriched (Benjamini-Hochberg adjusted *p* value < 0.05) at day 2, 3, 8, 30 and 36 in vaccinated subjects compared to placebo controls. Colour intensity and size of the dots is proportional to the −log10 transformed Benjamini-Hochberg adjusted *p* values. **g** Clustergram showing the log2-transformed fold-change of DEGs present in the top two BTM modules highlighted in **f**, “Enriched in monocytes (II) (M11.0)” and “Regulation of antigen presentation and immune response (M5.0)” at day 2, 3, 8, 30 and 36. The colour-gradient from blue to red indicates log2-transformed fold change (day 2/day 1) values from −2 to 2 respectively.

Total RNA was extracted from the whole blood of 106 healthy volunteers who provided written informed consent for participation in a phase I/II randomised, placebo-controlled clinical trial that assessed the safety and immunogenicity of ARCT-021 (clinicaltrial.gov NCT04480957). Gene transcript levels were determined at baseline (pre-dose 1), day 2, 3, and 8 for all vaccinees. In addition, gene expression was profiled at day 29 (pre-dose 2), 30 and 36 among vaccinees in the two-injection cohort. The magnitude of antibody responses to ARCT-021 was measured by the Luminex immuno-assay against the full-length recombinant S protein, and the S-reactive T-cell responses were assessed using IFNγ ELISPOT assay following stimulation with overlapping S protein peptide pools. The effects of vaccine dose and subject demographics on antibody and T-cell responses has been previously reported^15^. Among the ARCT-021 vaccinees, there was variation in S-binding IgG titers (Fig. 1a) and T-cell IFNγ responses (Fig. 1b) of >100 fold. We found low or weak correlation between S-binding IgG titers and T-cell IFNγ responses (Fig. 1c), although both measurements approximate to a Gaussian distribution (Supplementary Fig. 1a). These observations provided us with an opportunity to dissect the molecular signatures driving humoral and cell-mediated immunity to sa-mRNA vaccination.

### Early innate immune responses to ARCT-021

To examine the early innate immune gene signatures induced by ARCT-021, we conducted NanoString profiling using the nCounter Human Immunology v2 Panel (NanoString Technologies), which allows quantification of 579 immune-related transcripts with high precision^16^. The greatest number of differentially expressed genes (DEGs) (fold change >1.3; false discovery rate [FDR]-adjusted *p*-value < 0.05, Benjamini-Hochberg step-up procedure) was detected at day 2, and the number of DEGs decreased three-fold at day 3 (Fig. 1d). Secondary vaccination generated similar changes in transcriptional responses at day 30 relative to day 2 (Fig. 1d), with strong correlation observed for expression of transcripts between these 2 time points (Supplementary Fig. 1b). Notably, the most upregulated genes comprised cytokines, chemokines and interferon (IFN)-stimulated genes, including CXCL10, CCL2, CCL8, LAMP3, IFIT2 and MX1 (Fig. 1e, Supplementary Fig. 1c). Visualisation of gene networks by Ingenuity Pathway Analysis (IPA) revealed a tight interacting network of transcription factors responsible for the induction of these cytokines and chemokines, including IRF3, IRF7, STAT-1 and STAT-2 (Supplementary Fig. 2).

To dissect the molecular pathways induced by ARCT-021, we utilised the blood transcription modules (BTMs), a curated database comprising of an integrated large-scale network of publicly available human blood transcriptomes^17^. We found that BTMs related to activation of myeloid cells, antigen presentation, cell cycle and interferon signalling were significantly enriched on days 2, 3 and 30. The majority of transcripts returned to baseline levels at day 8 and day 36 (Fig. 1d, f-g). The upregulated transcripts in the enriched in monocytes (II) (M11.0) module included Toll-like receptors (TLR1-8), leukocyte immunoglobulin-like receptors and inflammatory mediators (Fig. 1g, Supplementary Fig. 3a), indicating that ARCT-021 vaccination leads to an early activation of innate immune responses. Furthermore, the majority of upregulated transcripts related to antigen presentation constitute the MHC-I signalling pathway, including induction of transcripts involved in peptide degradation and processing (LMP2, LMP7), peptide loading (TAP1, TAP2, TPN, CLIP) and MHC-I molecules, which could facilitate antigen presentation to CD8 T-cells (Supplementary Fig. 3b). Taken together, our data showed that vaccination with ARCT-021 triggers an early and strong induction of the innate immune response, leading to upregulation of genes involved in pattern recognition receptor signalling, cytokine and chemokine signalling and MHC-I antigen presentation.

### Expression of BSIG transcripts 1 day post-vaccination correlates with humoral responses to ARCT-021

**Fig. 2:**
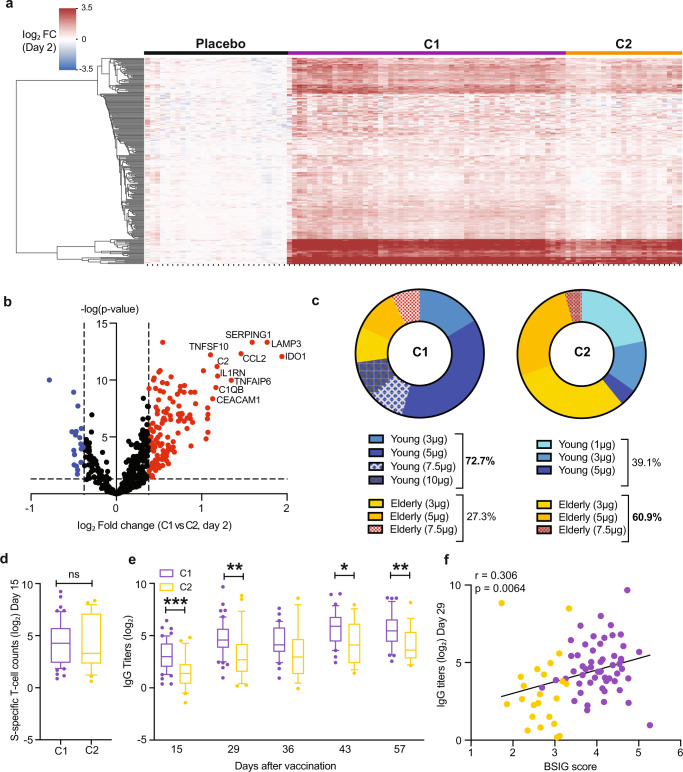
Magnitude of induction of BSIG transcripts is positively correlated with humoral responses. **a** Gene and sample hierarchical clustering based on expression profiles of upregulated DEGs on day 2. Three distinct sample clusters, named placebo, C1 (purple) and C2 (yellow) are detected. The colour-gradient from blue to red indicates log2-transformed fold change (day 2/day 1) values from −3.5 to 3.5 respectively. **b**. Volcano plot displaying genes that were most differentially expressed between C1 and C2 on day 2. The top 10 most differentially regulated genes are annotated on the volcano plot. Fold-change cut-off of 1.3 and Benjamini-Hochberg adjusted p-value 0.05 are indicated as dotted lines on the volcano plot. **c**. Donut plots showing the demographics of C1 and C2. Young and elderly subjects are highlighted in blue and yellow-orange respectively, with higher doses of vaccines indicated in darker colours. Overall percentages in the young and elderly subjects in C1 and C2 are also displayed. **d**. S-specific T-cell responses at day 15 post-vaccination in C1 (*n* = 55) and C2 (*n* = 23) clusters. **e**. Anti-S IgG titers (log_2_-transformed) of subjects in clusters C1 and C2 at day 15, 29, 36, 43 and 57 post-vaccination. For time-points from day 36 onwards, analysis is based on subjects who received the second dose of the vaccine. Box plots in **d-e** represent 25–75% intervals, with lines indicating medians. The whiskers represent 10–90% intervals. Unpaired, two-sided, Student’s t-tests were used for comparisons for **d-e**. **f** Pearson correlation of log_2_-transformed IgG titers at day 29 with BSIG score. BSIG score is calculated as the arithmetic mean of the top 10 genes that most distinctly separate C1 and C2, as indicated in **b**. *P < 0.05, **P < 0.01, ***P < 0.001.

An unsupervised hierarchical clustering of the 231 upregulated DEGs at day 1 post-ARCT-021 vaccination indicated 3 distinct clusters: placebo group, C1 (purple) and C2 (yellow) (Fig. 2a). The top 10 differentially regulated genes between C1 and C2 were related to complement activation (C1QB, C2, SERPING1) and inflammatory response (CCL2, IL1RN, IDO1, TNFAIP6) (Fig. 2b, Supplementary Fig. 4a-b). The majority of participants in C1 were younger adults who received at least 3 µg of ARCT-021 (Fig. 2c). In contrast, participants in C2 consisted of either older adults or those who received lower doses (1µg-3µg) of ARCT-021 (Fig. 2c), indicating that the transcriptional responses to ARCT-021 in older adults are more blunted.

Next, we examined if the C1-C2 dichotomy affected anti-S IgG titers and S-specific T-cell IFNγ responses. While no significant differences were seen in T-cell responses (Fig. 2d, Supplementary Fig. 4c), anti-S IgG titers at day 15, 29, 43 and 57 were significantly lower in C2 compared to C1 (Fig. 2e, Supplementary Fig. 4d). To verify that the gene expression differences between C1 and C2 were indeed correlated with anti-S IgG titers, we calculated the arithmetic mean of the log2 fold change values from the top 10 transcripts that most distinctly separate C1 and C2 (termed as BSIG score) for each sample, and performed a correlation analysis with anti-S IgG titers. Indeed, the BSIG score was significantly correlated with anti-S IgG titers (Fig. 2f), thus supporting an association between the magnitude of induction of the BSIG transcripts at 1 day post-vaccination with humoral responses to ARCT-021.

A random forest regression model (Supplementary Fig. 5a) was used to identify the immune transcripts that could most accurately predict anti-S IgG titers. 75% of the samples were used as training data and the remaining 25% as test data to evaluate the accuracy of our machine learning model. Our first model generated a decision tree that was able to predict anti-S IgG titers with 84.8% accuracy, with root mean square error (RMSE) of 1.43. The relative feature importance of the individual genes involved in generation of the decision tree is as depicted in Supplementary Fig. 5b. After hyperparameter tuning, we ascertained that the random forest regression model based on the log2 fold-change values of the top 6 genes (KLRB1, CCL2, FCER1G, ITGA6, MSR1 and GPR183) further improved the accuracy to 96.0% (RMSE = 1.70) (Supplementary Fig. 5c). Notably, many of these genes are involved in leukocyte migration (GO:0060900, FDR = 0.0179), highlighting the pivotal role of chemotaxis in generation of humoral immune responses to ARCT-021.

Since the magnitude of BSIG upregulation is associated with higher antibody response, we further examined upregulated immune transcripts that best differentiate responders from non-responders. Responders were defined by subjects that seroconverted with at least a 4-fold rise in antibody titers^15^. In agreement with the random forest regression model (Supplementary Fig. 5b), we identified MSR1 and FCER1G to be most significantly increased in responders (Supplementary Fig. 6a-c). Both MSR1 and FCER1G were also significantly increased in responders compared to non-responders among the older adults (Supplementary Fig. 6d). However, the magnitude of upregulation for MSR1 and FCER1G was not associated with severity of side effects (Supplementary Fig. 6e-f). Likewise, BSIG score was not found to be associated with severity of side effects (Supplementary Fig. 6g), demonstrating that the transcriptomic signatures for ARCT-021 immunogenicity and reactogenicity are distinct.

### Immune signatures at day 8 correlated with spike-specific T-cell response to ARCT-021

**Fig. 3:**
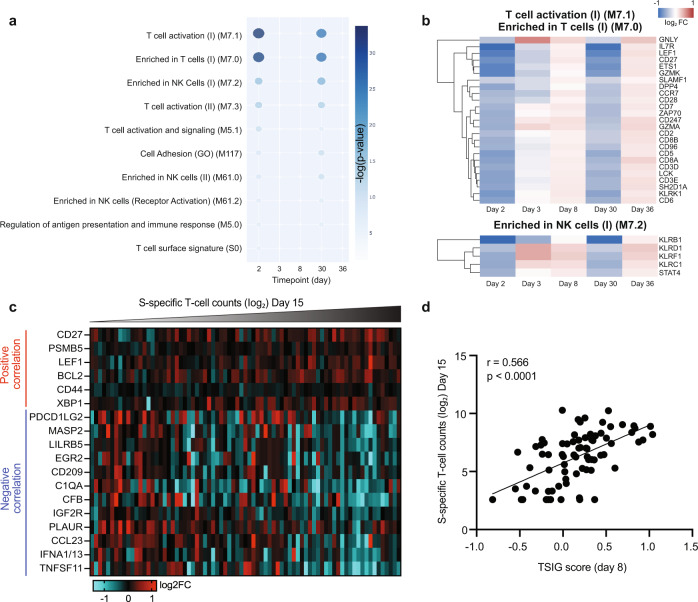
Transcriptional correlates of spike-specific T cell responses are observed at day 7. **a** Top 10 blood transcription modules (BTMs) that are negatively enriched (Benjamini-Hochberg adjusted *p*-value < 0.05) at day 2, 3, 8, 30 and 36 in vaccinated subjects compared to placebo controls. Colour intensity and size of the dots are proportional to the −log10 transformed Benjamini-Hochberg adjusted *p*-values. **b** Clustergram showing the log2-transformed fold-change of DEGs present in the top BTM modules highlighted in, “T-cell activation (I) (M7.1)”, “Enriched in T cells (I) (M7.0)” and “Enriched in NK cells (I) (M7.2)” at day 2, 3, 8, 30 and 36. The colour-gradient from blue to red indicates log2-transformed fold change (day 2/day 1) values from −1 to 1 respectively. **c** Heatmap showing day 8 transcripts that are significantly correlated (*p*-value < 0.05) with log2-transformed S-specific T cell counts at day 15 in ARCT-021 vaccinated individuals. Transcripts are ranked by Pearson correlation coefficient values. **d**. Pearson correlation of log2-transformed S-specific T cell counts at day 15 with TSIG score. TSIG score for each sample was calculated by subtracting the arithmetic log2 fold change values of the 12 negatively correlated genes from the 6 positively correlated genes shown in panel c.

Next, we performed pathway enrichment for downregulated genes following ARCT-021 vaccination. Negatively enriched BTMs were mostly related to T-cells and NK cells (Fig. 3a), with genes that encode for surface receptors on T-cells and NK cells being significantly downregulated at day 2 and day 30 (Fig. 3b). However, expression of downstream T-cell signalling transcripts remained unchanged (Supplementary Fig. 7), suggesting that ARCT-021 vaccination did not lead to global suppression of T-cell activity. Instead, the downregulation of these T-cell transcripts could indicate increased migration of T-cells out of the peripheral blood, and likely to lymphoid organs. In addition, the majority of downregulated NK cell-related genes encoded for inhibitory receptors (KLRB1, KLRC1, KLRD1), suggesting an early activation of NK cells post-vaccination (Fig. 3b). To ascertain if the differences in downregulated DEGs post-vaccination could predict differences in T-cell responses, we performed an unsupervised hierarchical clustering, which generated 3 distinct clusters: placebo group, T1 (blue) and T2 (pink) (Supplementary Fig. 8a). However, the T1-T2 dichotomy was unable to discriminate subjects based on vaccine-induced T-cell response (Supplementary Fig. 8b). Instead, gene expression signatures at day 8 identified transcripts that were associated with T-cell responses. Interestingly, a total of 18 transcripts (6 positively correlated, 12 negatively correlated) were found to be significantly correlated with CD8 T-cell responses (Fig. 3c). We calculated a TSIG score for each sample by subtracting the arithmetic log2 fold change values of the 12 negatively correlated genes from that of the 6 positively correlated genes. The TSIG scores were significantly correlated with T-cell response, with correlation coefficient of 0.566 (*p* < 0.0001) (Fig. 3d), indicating that the expression levels of these 18 transcripts at day 8 could discriminate subjects with strong from weak T-cell response. Of note, many of the positively correlated transcripts were associated with T-cell maturation and expansion (CD27, LEF1, CD44, XBP1), as well as proteasome degradation (PSMB5)^18–22^. Conversely, negatively correlated transcripts were mostly associated with complement activation (MASP2, C1QA, CFB), T-cell proliferation (PDCD1LG2), dendritic cell activity (TNFSF11, CD209, IFNA1/13) and type I interferon response (IFNA1/13).

Given that the top 3 positively correlated genes (CD27, LEF1 and PSMB5) were previously demonstrated to be critical for CD8 T-cell expansion, maturation and MHC-I presentation^18–20,22^, we examined if the random forest regression model based on log2 fold-change values of these genes could accurately predict spike-specific T-cell response. The accuracy of the model based on CD27, LEF1 and PSMB5 was found to be 70.43% (RMSE = 2.11) (Supplementary Fig. 8c), suggesting that the induction of these transcripts is important in inducing spike-specific T-cell responses.

### Comparison of ARCT-021 with other vaccines

To understand how the host response to sa-mRNA vaccination fares against those to other vaccines, we performed a comparative analysis of our data with other published vaccine trials (Supplementary Table 1). Immune-related genes induced by different vaccines were visualized using a correlation matrix showing pairwise correlations of the mean gene log2-transformed fold changes between vaccines. When we compared gene expression at 1 day post-vaccination, ARCT-021 exhibited a strong correlation with live viral vectors (MRKAd5-HIV and rVSV-ZEBOV vaccines), adjuvanted vaccines (HepB + AS01B/AS01E and H5N1 + AS03) and mRNA vaccines (BNT162b2). However, little overlap was seen with the live-attenuated vaccines, unadjuvanted (H5N1) and polysaccharide vaccines (Pneumovax23) (Supplementary Fig. 8d). Closer examination of the host response kinetics for each vaccine showed that the peak transcriptional responses for the live-attenuated vaccines occurred at later time-points, at day 7 post-vaccination as compared to other vaccines^10,23–25^, which occurred at day 1 post-vaccination.

**Fig. 4:**
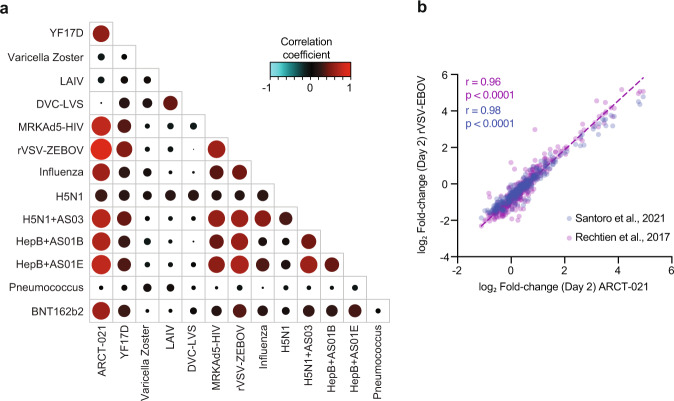
ARCT-021 gene signatures are correlated with live viral vectors, adjuvanted and mRNA vaccines. **a** Correlation matrix showing pairwise correlations of the mean gene log2-transformed fold changes between the different vaccines at time-points with peak transcriptional responses. Transcriptional responses peaked on day 7 for live-attenuated vaccines and Pneumovax, day 3 for H5N1 and day 1 for all other vaccines. Size and intensity of dots are proportional to the magnitude of correlation coefficient. **b** Pearson correlation of log2-transformed fold-changes of transcripts in subjects receiving rVSV-ZEBOV vs ARCT-21 at day 1 post-vaccination^26,27^. r = correlation coefficient, p = significance of correlation.

To investigate if the weak correlation with live-attenuated vaccines was attributed to differences in kinetics of vaccine-induced host response, we visualized the correlation matrix at time-points where peak transcriptional responses were observed for each vaccine. Indeed, the correlation with YF17D was much stronger (Fig. 4a), suggesting that the transcriptional alterations in YF17D were more prolonged and delayed as compared to ARCT-021. Among all the vaccines analysed, rVSV-ZEBOV displayed the strongest correlation with ARCT-021, and this finding was consistent across 2 different studies^26,27^ (Fig. 4b). Collectively, these observations suggest that a sa-mRNA vaccine that is able to induce the innate immune responses observed albeit in a more delayed fashion could potentially engender immunogenicity that approaches those of LAVs.

## Discussion

The innate immune response to vaccination plays an important role to stimulate the adaptive immune response that provides the specificity in protecting against infectious diseases. Detailed understanding of the types of innate immune responses induced by vaccination and their expression kinetics could thus be used to guide further development or refinement of new vaccines.

Our findings reveal that the peak transcriptional changes in innate immune genes to ARCT-021 vaccination were observed at day 2. The magnitude of the induction of innate immune responses, including genes driven by monocytes, dendritic cells, TLR signalling, inflammatory responses, complement activation and chemokines to ARCT-021 correlated positively with anti-S IgG titers at day 29. Notably, a greater induction of innate immune responses involving monocytes, TLR and inflammatory signalling was also associated with higher antibody titers following the BNT162b2 mRNA booster vaccination, and adjuvanted hepatitis B vaccination in humans^11,28^, indicating the importance of these pathways in inducing robust antibody responses.

Most of the previous studies on the innate immune response to vaccination have focused on identifying correlates of antibody response; few have focused on the correlates of cellular immune responses. Given the important role cellular immunity plays in protection against COVID-19, we also probed the correlates of T cell responses from ARCT-021 vaccination. Unlike the correlates of antibody response, correlates of T-cell responses were apparent only at day 8, with genes related to T-cell maturation correlating positively with S-reactive T-cell responses at day 15. In addition, there was also significant induction of transcripts related to peptide degradation and processing, peptide loading and MHC class I, which can in turn promote CD8 T-cell survival and responses^29^. Two possible reasons may account for why T-cell signatures were more apparent at a later time point. One possibility is that the early migration of T-cells from peripheral blood to lymph nodes may have masked the T-cell proliferation, activation and maturation signatures, which was also observed in SARS-CoV-2 infected individuals^30,31^ and in individuals who received the influenza vaccine^32^. The other possibility is a lag phase between ARCT-021 vaccination and induction of T-cell signatures. Indeed, T-cell signatures in live-attenuated vaccines (YF17D, LAIV)^8,24^, MRKAd5/HIV^33^ and adjuvanted vaccines^34^ were only induced 3 days after vaccination. Moreover, memory CD8 T-cells have been shown to reside primarily in the lymphoid organs^35^, which may have hindered the detection of transcripts related to T-cell memory. Future studies that evaluate gene expression in tonsils or lymph nodes after sa-mRNA vaccination could thus be insightful.

Although the early transcriptomic signatures of BNT162b2 and ARCT-021 were largely similar after the first dose, an interesting observation was that the innate immune activation was not of a higher magnitude after the second dose of ARCT-021. This is in contrast to BNT162b2^11^ and adjuvanted vaccines^28^ where the second dose resulted in greater innate immune activation. A plausible explanation could be that a single dose of the sa-mRNA vaccine is sufficient to induce significant up- or down-regulation of immune genes as self-replication results in double-stranded RNA intermediates with immunostimulatory properties that can likely prolong and intensify the ensuing innate immune response. However, over-activation of innate immune response may block translation and amplification of sa-mRNA, and potentially reduce vaccine immunogenicity. We observed that the initial stimulation of innate immune response following ARCT-021 vaccination resolves by day 8. Moreover, downregulation of interferon-alpha at day 8 was associated with better T cell responses.

In addition, our findings also suggest the potential of sa-mRNA vaccines to be further engineered to elicit the innate immune responses and consequently the immunogenicity associated with LAVs. Indeed, the association of sa-mRNA signatures with the YF17D day 7 signatures, as well as the delayed expression of the correlates of T cell responses at day 8 suggests that a longer period of antigen presentation may favour more robust B and T cell development. This could be achieved by introducing mutations in the sequence of non-structural proteins encoding the replicase to either slow down mRNA replication kinetics or through sequence optimization of 5’ and 3’ untranslated regions that may alter RNA secondary structures to avoid prematurely triggering the host immune response for enhanced translation of the sa-mRNA^36,37^. Consequently, further investigation of the interactions between sa-mRNA and host proteins would be warranted to strategically avoid a sharp but short surge of innate immune activity to prolong sa-mRNA replication and translation for enhanced antigen presentation and vaccine immunogenicity.

In conclusion, we identified gene expression signatures that correlated with humoral and T-cell responses to ARCT-021 vaccination and showed that sa-mRNA is able to trigger the innate immune responses observed in viral vector vaccines, AS01 and AS03 adjuvanted vaccines as well as BNT162b2. The molecular insights underpinning induction of adaptive immune response to ARCT-021 can guide the development of next-generation sa-mRNA vaccines for improved immunogenicity.

## Methods

### Clinical trial design

ARCT-021-01 is a randomized, double-blinded, placebo (0.9% saline) controlled study, evaluating a range of doses of ARCT-021 relative to placebo in younger (21 to 55 years) and older (56 to 80 years) adult participants^15^. A total of 106 healthy participants were randomized into single and two-injection cohorts. In single injection cohorts, ARCT-021 was given as an intramuscular injection to younger (1 µg, 5 µg, 7.5 µg and 10 µg ARCT-021 versus placebo) and older adults (7.5 µg ARCT-021 versus placebo). In two-injection cohorts, two doses of ARCT-021 (3 µg and 5 µg ARCT-021 versus placebo) were administered 28 days apart to younger and older adults.

### Immunogenicity assessments

Luminex immuno-assays were conducted to measure IgG against full-length recombinant spike protein at indicated time points following vaccination. Full-length recombinant SARS-CoV-2 spike protein (AcroBiosystems) was conjugated to Magpix Luminex beads using the ABC coupling kit (Luminex). Spike protein conjugated beads were blocked with 1% BSA in PBS with 0.05% Tween 20 and incubated with sera for 1 hour at 37 °C. Beads were then washed and incubated with PE-conjugated anti-human IgG secondary antibody (Invitrogen) for 30 minutes at 37 °C. Spike-binding antibodies were measured as median fluorescence intensity (MFI) using a Magpix instrument. SARS-CoV-2 specific T-cell responses were assessed using IFNγ ELISPOT assay following stimulation with overlapping S protein peptide pools. ELISpot plates were coated with human IFNγ antibody (Mabtech) overnight at 4 °C. 200,000 PBMCs were seeded in each well and stimulated overnight with the S protein peptide pools. Plates were incubated with human biotinylated detection mAb (7-B6-1) before streptavidin-ALP was added. Plates were developed using BCIP/NBT phosphatase substrate (Seracare) and results expressed in spot forming cells (SFC)/10^6^ PBMCs.

### NanoString profiling

NanoString profiling was performed using the nCounter Human Immunology v2 Panel (NanoString Technologies, Inc. Cat# XT-CSO-HIM2-12). 50 ng of RNA was mixed with 8 ul of reporter probeset and 2ul of capture probeset, and incubated at 65 °C for 24 h. Hybridised samples were loaded onto an nCounter Sprint cartridge and imaged on a nCounter Sprint system. Raw and normalised counts were derived with the NanoString nSolver 4.0 software as per manufacturer’s protocols. nCounter probe sets included negative and positive controls that were used for background thresholding, and normalizing samples for differences in sample input or hybridisation.

### Transcriptomics analysis

To identify DEGs, gene expression values were compared between vaccinated and placebo subjects. DEGs were identified based on fold-change >1.3 with respect to the placebo controls at the respective days, with false discovery rate adjusted *p*-value < 0.05 using the Benjamini-Hochberg step-up FDR-controlling procedure in Partek® Genomics Suite®. For pathway analysis, the identified DEGs were used as input data, and analysed against the blood transcriptomics modules (BTMs) database^17^. Enriched pathways were ranked and identified based on adjusted *p*-values < 0.05, using GSEApy (https://github.com/ostrokach/gseapy), a python wrapper for GSEA and Enrichr^38^. Ingenuity pathway analysis was used to detect the gene networks and pathways that are differentially regulated after ARCT-021 vaccination. Unsupervised hierarchical clustering and heatmaps were plotted using Seaborn, which is a Python data visualisation library based on matplotlib. BSIG scores for each subject were tabulated by calculating the arithmetic mean of the log2-fold change values (day 2 vaccinated versus placebo controls) of IDO1, LAMP3, SERPING1, CCL2, IL1RN, TNFAIP6, C1QB, CEACAM1, TNFSF10 and C2, which are the top 10 genes that are most differentially expressed between C1 and C2 groups. TSIG scores for each sample was calculated by subtracting the arithmetic mean of the log2 fold change values of the 12 negatively correlated genes from the 6 positively correlated genes.

### Analysis of gene expression and adverse events

Adverse events (AEs) were classified by system organ class according to the Common Terminology Criteria for Adverse Events (CTCAE) version 4.0, and graded as mild, moderate or severe^15^. For analysis of gene expression and side effects, subjects were grouped based on the highest grading of adverse events (AEs) reported. Subjects with no systemic AEs were scored as 0, mild AEs as 1, moderate AEs as 2 and severe AEs as 3.

### Vaccine dataset meta-analysis

Datasets were obtained from Gene Expression Omnibus (GEO) via accession identifiers shown in Supplementary Table 1. For MRKAd5-HIV dataset, host transcriptomics responses were only analysed for the seronegative subjects, as the vaccine did not protect against HIV acquisition in seropositive subjects^39^. CEL files were normalised and analysed with the Partek® Genomics Suite®. For RNAseq dataset, the normalised count matrix from GEO was used. Normalised log2 fold change values compared to baseline were computed for each vaccine, and the peak transcriptional responses were used for plotting of the correlation matrix. Correlation matrix was then visualised and plotted in R, using the cor() and corrplot() functions.

### Statistics

Differentially expressed genes (DEGs) were defined as genes with fold-change > 1.3 and False Discovery Rate [FDR]-adjusted *p*-value < 0.05, Benjamini-Hochberg step-up procedure in Partek Genomics Suite. Ranking and identification of enriched pathways was based on adjusted *p*-values < 0.05, using GSEApy. Unpaired, two-sided Student’s t-test was used for comparisons between vaccinated groups using Prism v.9.1 software (GraphPad Software Inc.).

### Study approval

This study was approved by the SingHealth Centralized Institutional Review Board (CIRB/F 2020/2553) and the Singapore Health Sciences Authority. The trial was registered at ClinicalTrials.gov (NCT04480957). Written informed consent was obtained from all participants before enrolment.

### Reporting summary

Further information on research design is available in the Nature Research Reporting Summary linked to this article.

## Supplementary information

Supplementary Information

REPORTING SUMMARY

## Data Availability

The raw data and log_2_ fold change values for NanoString profiling of immune responses are available at Array Express (E-MTAB-11315).
