# Supplementary material for: Immune gene expression analysis indicates the potential of a self-amplifying Covid-19 mRNA vaccine
Source: NPJ Vaccines. 2022 Nov 28;7:154. doi: 10.1038/s41541-022-00573-y (PMC9703414; doi:10.1038/s41541-022-00573-y)
Supplement: Supplementary file 1 — Supplementary Information [file 41541_2022_573_MOESM1_ESM.pdf]

Supplementary Fig. 1

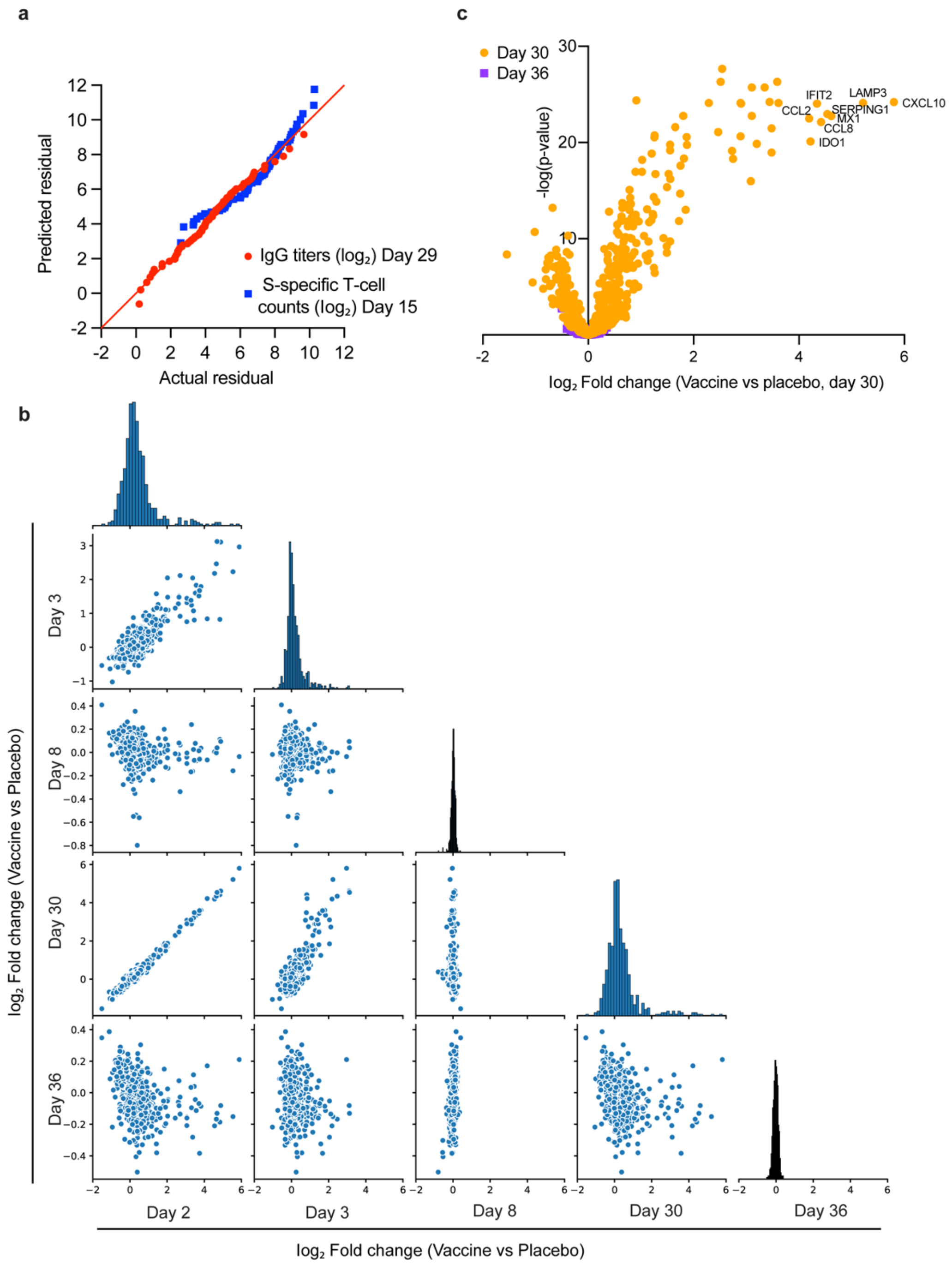

Supplementary figure 1. Day 2 and day 30 transcriptional responses are highly correlated. a. QQ-plot of the predicted residuals against actual residuals for antibody and T cell responses. Red line indicates that the data points follow a Gaussian distribution. **b.** Volcano plot displaying genes that were most differentially expressed at day 30 and 36. The most differentially regulated genes are annotated on the volcano plot. **c.** Pair plots illustrating the correlation of log2-transformed fold-changes between day 2, 3, 8, 30 and 36, in subjects who received ARCT-021 compared to the placebo controls.

Supplementary Fig. 2

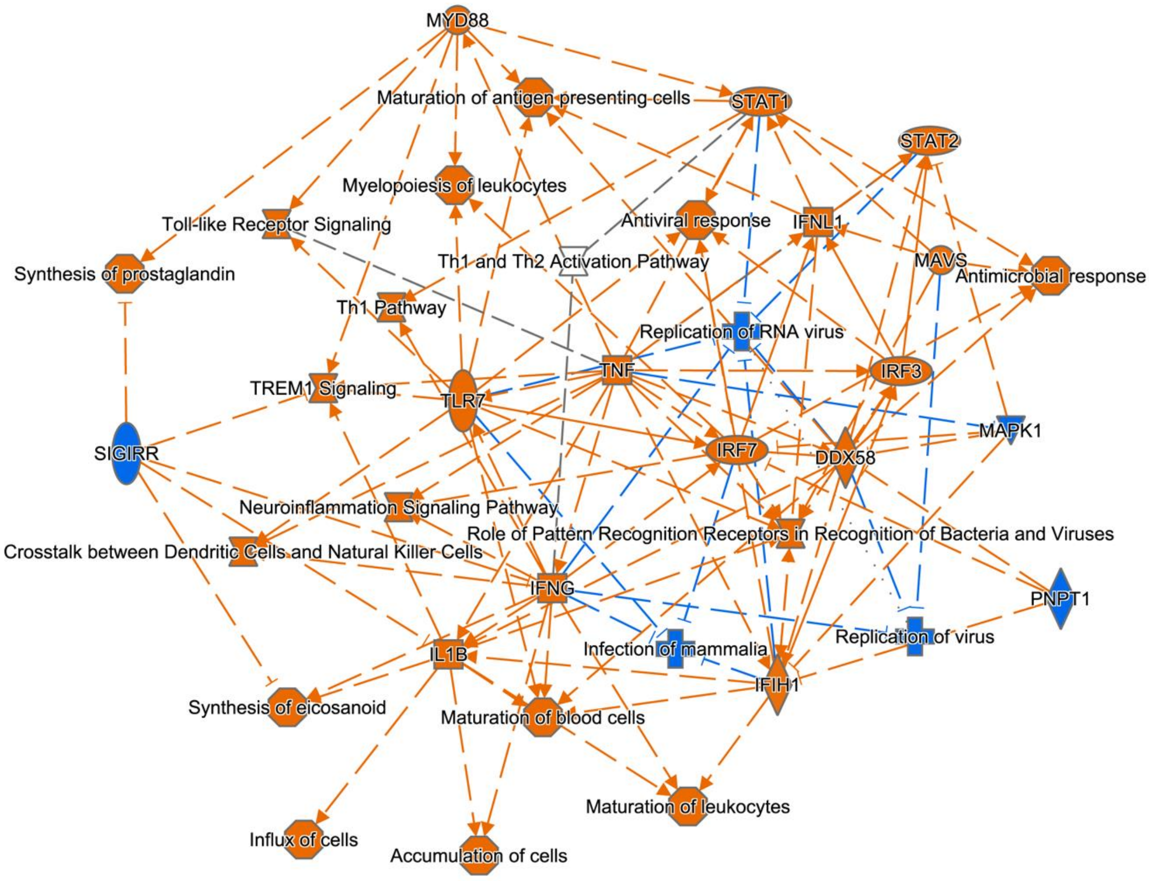

Supplementary figure 2. Gene regulatory networks that are induced by ARCT-021 one day following vaccination. Ingenuity pathway analysis of a subset of genes that are identified as being significantly upregulated on day 2 (False Discovery Rate [FDR]-adjusted p-value < 0.05, Benjamini-Hochberg step-up procedure).

Supplementary Fig. 3

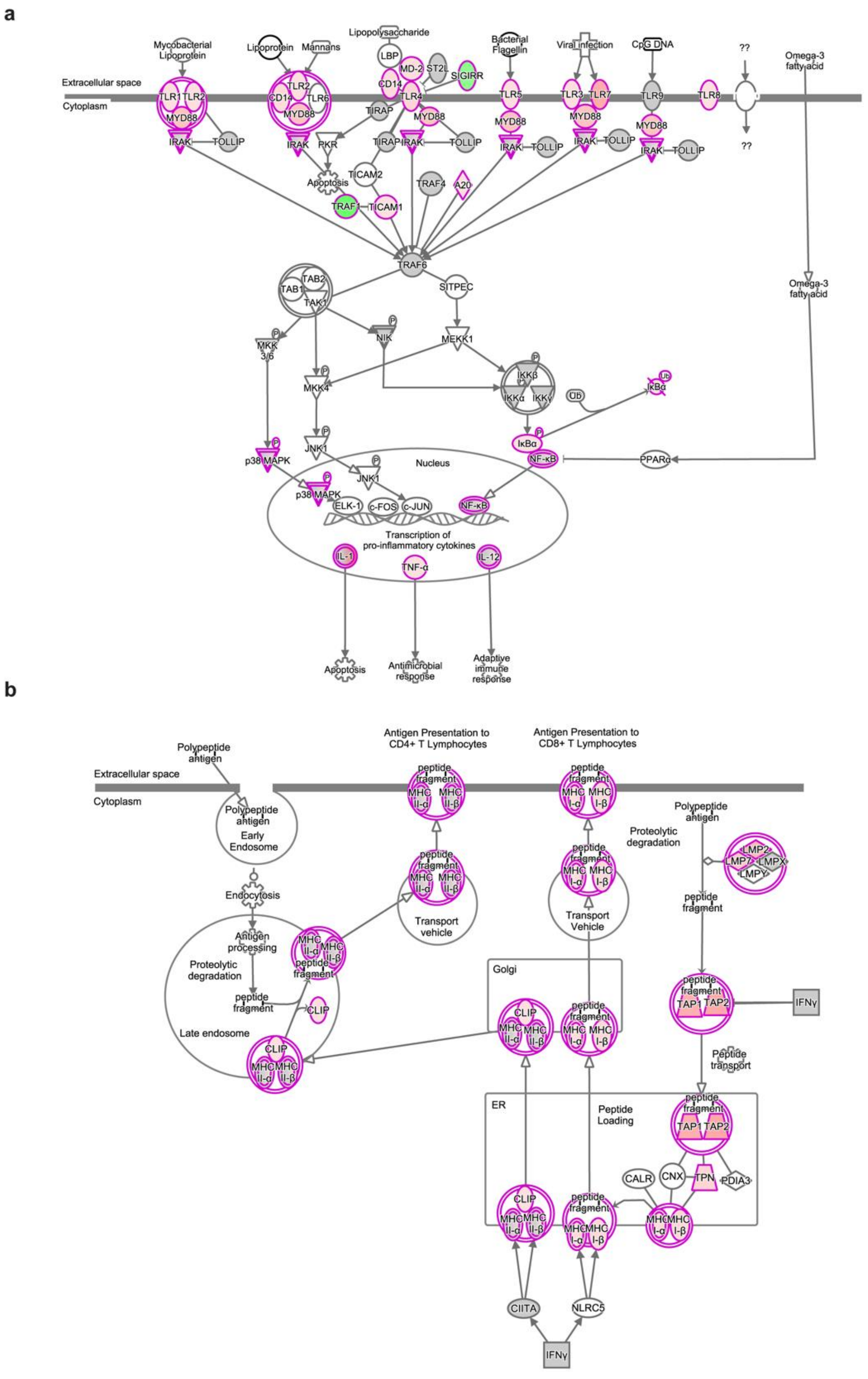

Supplementary figure 3. Transcripts related to pattern recognition receptor signalling and MHC-I antigen presentation are upregulated one day following vaccination. Ingenuity pathway analysis showing transcripts involved in **a.** pattern recognition receptor signaling and **b.** antigen presentation that are significantly changed on day 2 (False Discovery Rate [FDR]-adjusted p-value < 0.05, Benjamini-Hochberg step-up procedure). Pink ovals indicate transcripts that are upregulated whereas green ovals indicate transcripts that are downregulated.

Supplementary Fig. 4

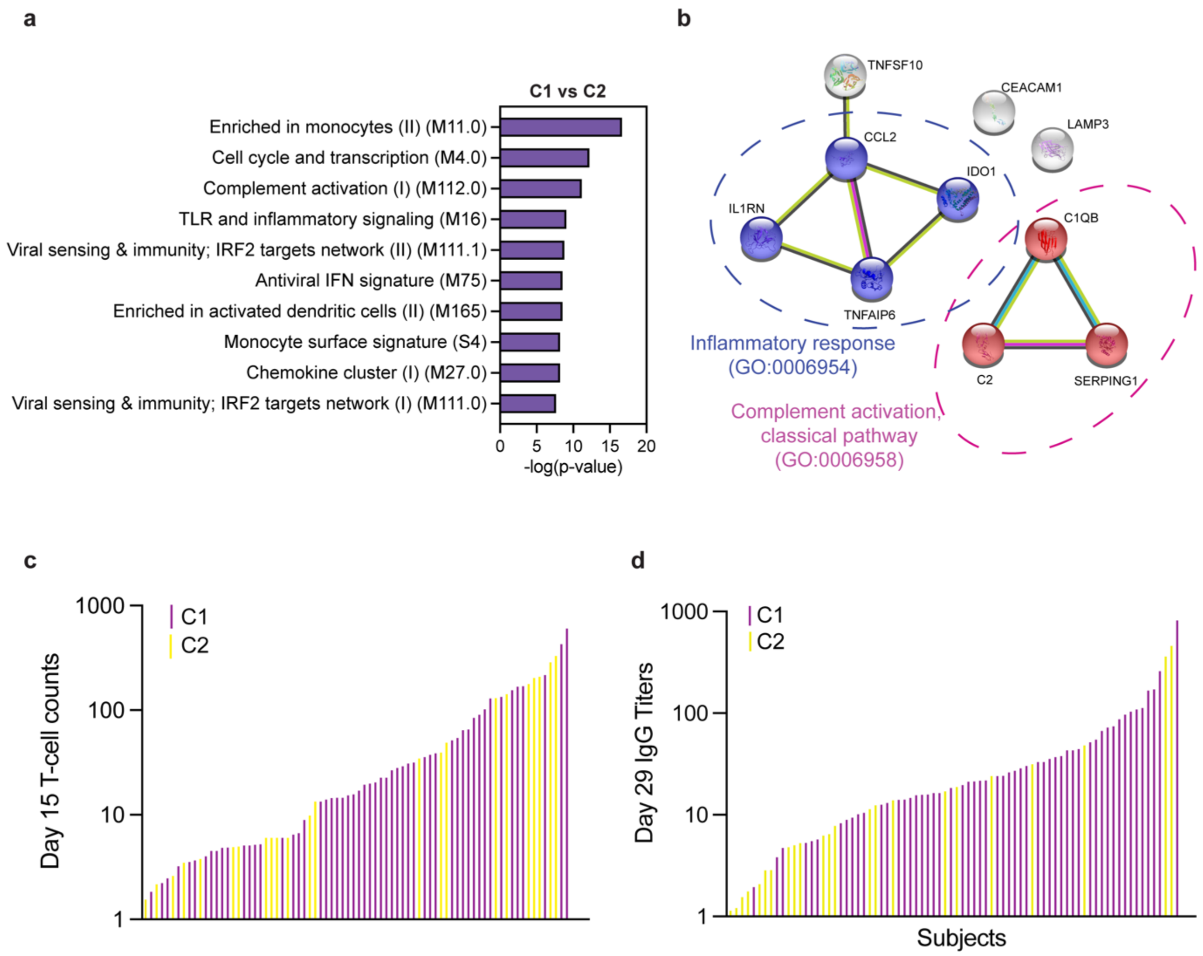

Supplementary figure 4. Transcripts and enriched gene sets that best distinguish C1 from C2 subjects. a. Top 10 blood transcription modules (BTMs) that are most significantly enriched in C1 compared to C2 based on differentially expressed genes (DEGs). DEGs were defined as genes with fold-change > 1.3 and False Discovery Rate [FDR]-adjusted p-value < 0.05, Benjamini-Hochberg step-up procedure. **b.** GO biological processes enriched among the BSIG genes. **c.** Day 29 IgG antibody titers and **d.** Day 15 S-specific T cell responses in vaccinated individuals, ranked from smallest to largest values and coloured by C1 (purple) or C2 (yellow) group.

Supplementary Fig. 5

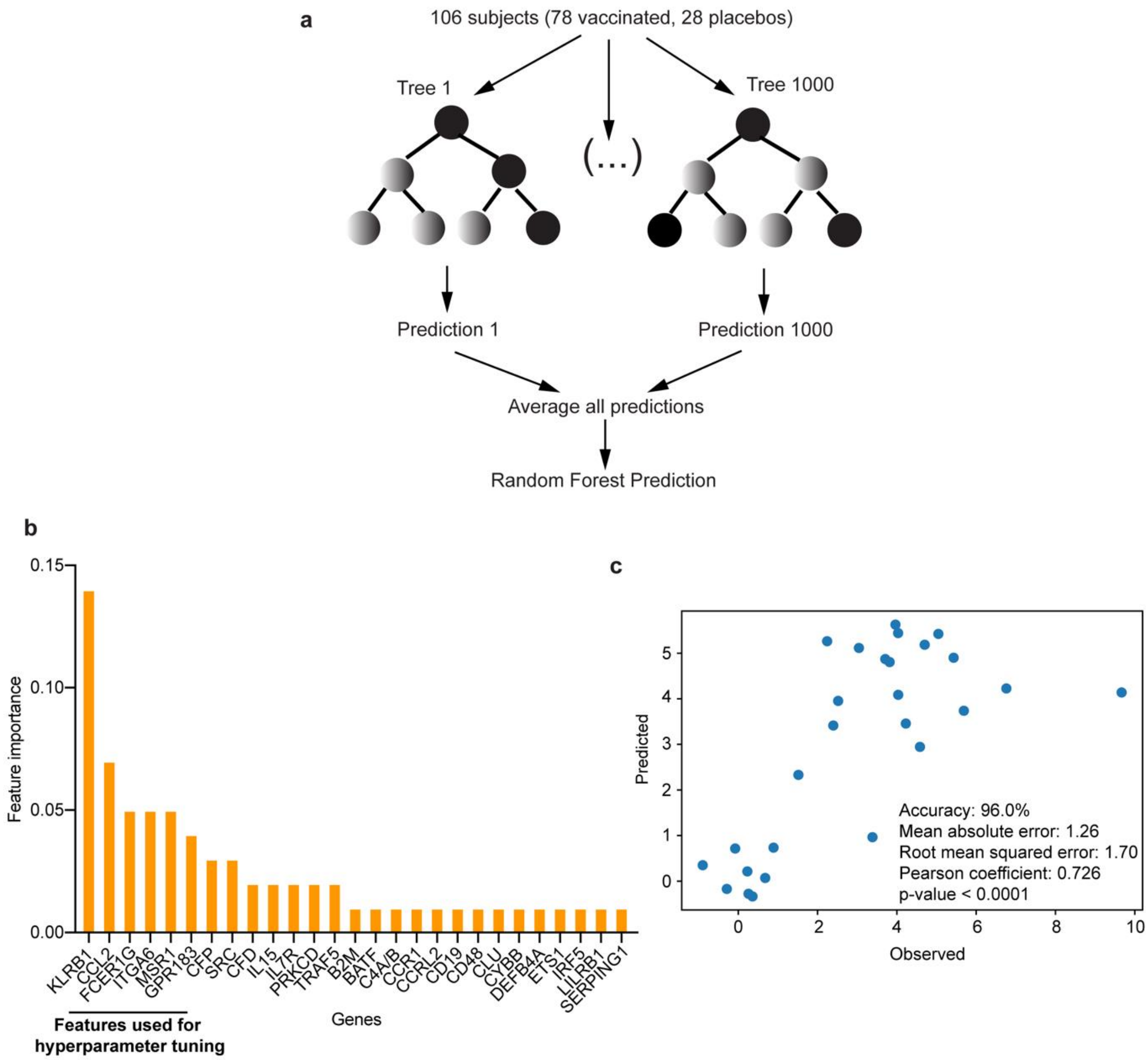

Supplementary figure 5. Random forest regression identifies transcripts that are most predictive of antibody responses to ARCT-021. a. Schematic of random forest regression. Machine learning is based on 106 subjects enrolled into the trial using random forest regression, with 75% of the subjects grouped into the training data and 25% of subjects grouped into test data. Final model was predicted based on averaging of 1,000 decision trees. **b.** Feature importance of the individual transcripts that were predictive of IgG titers at day 29. The top 6 genes were then selected for hyperparameter tuning, to further refine the random forest regression model. **c.** Scatterplot of the predicted and observed antibody titers, where predicted values were calculated based on the random forest regression model refined in **b**. Accuracy, mean absolute error, root mean-squared error, Pearson coefficient and p-values of the relationship between predicted and observed values are also displayed.

Supplementary Fig. 6

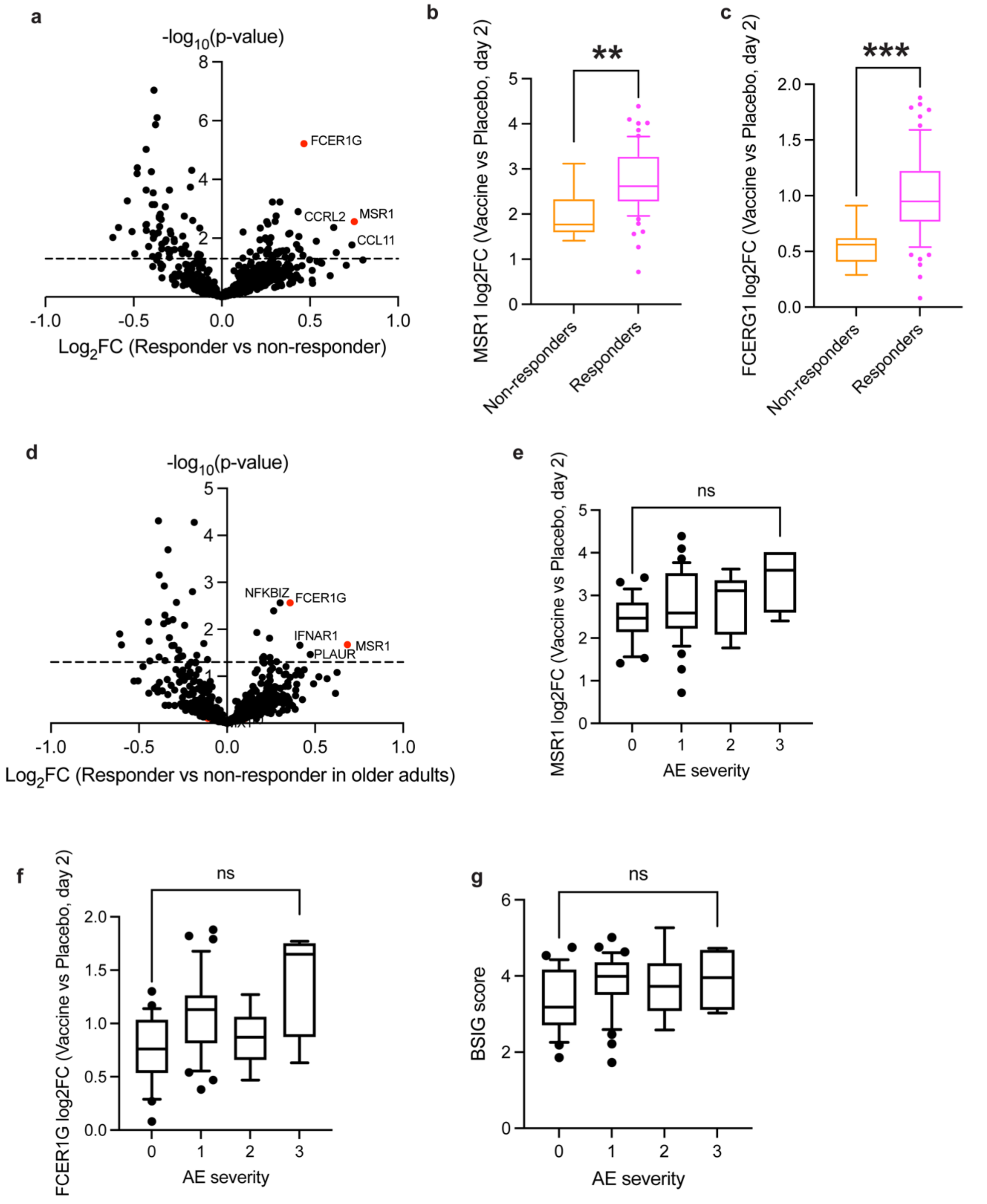

Supplementary figure 6. Expression of transcripts that best distinguish responders from non-responders are not associated with severity of adverse events (AEs). a. Volcano plot displaying genes that were most differentially expressed at day 2 after vaccination in responders relative to non-responders. The most differentially regulated genes are annotated on the volcano plot. **b**. Expression of MSR1 and **c**. FCERG1 on day 2 is significantly higher in responders than non-responders. Box plots in **b-c** represent 25%-75% intervals, with lines indicating

medians. The whiskers represent 10%-90% intervals. Unpaired, two-sided, Student's t-tests were used for comparisons for **b-c**. **d.** Volcano plot displaying genes that were most differentially expressed at day 2 after vaccination in responders relative to non-responders in older adults. The most differentially regulated genes are annotated on the volcano plot. **e**. Expression of MSR1 and **f**. FCERG1 on day 2 is not significantly different in subjects with varying levels of adverse event severity. Subjects with no systemic AEs were scored as 0, mild AEs as 1, moderate AEs as 2 and severe AEs as 3. **g**. BSIG score is not significantly different in subjects with varying levels of adverse event severity. Box plots in **e-g** represent 25%-75% intervals, with lines indicating medians. The whiskers represent 10%-90% intervals. Unpaired, two-sided, Student's t-tests were used for comparisons for **e-g**.

Supplementary Fig. 7

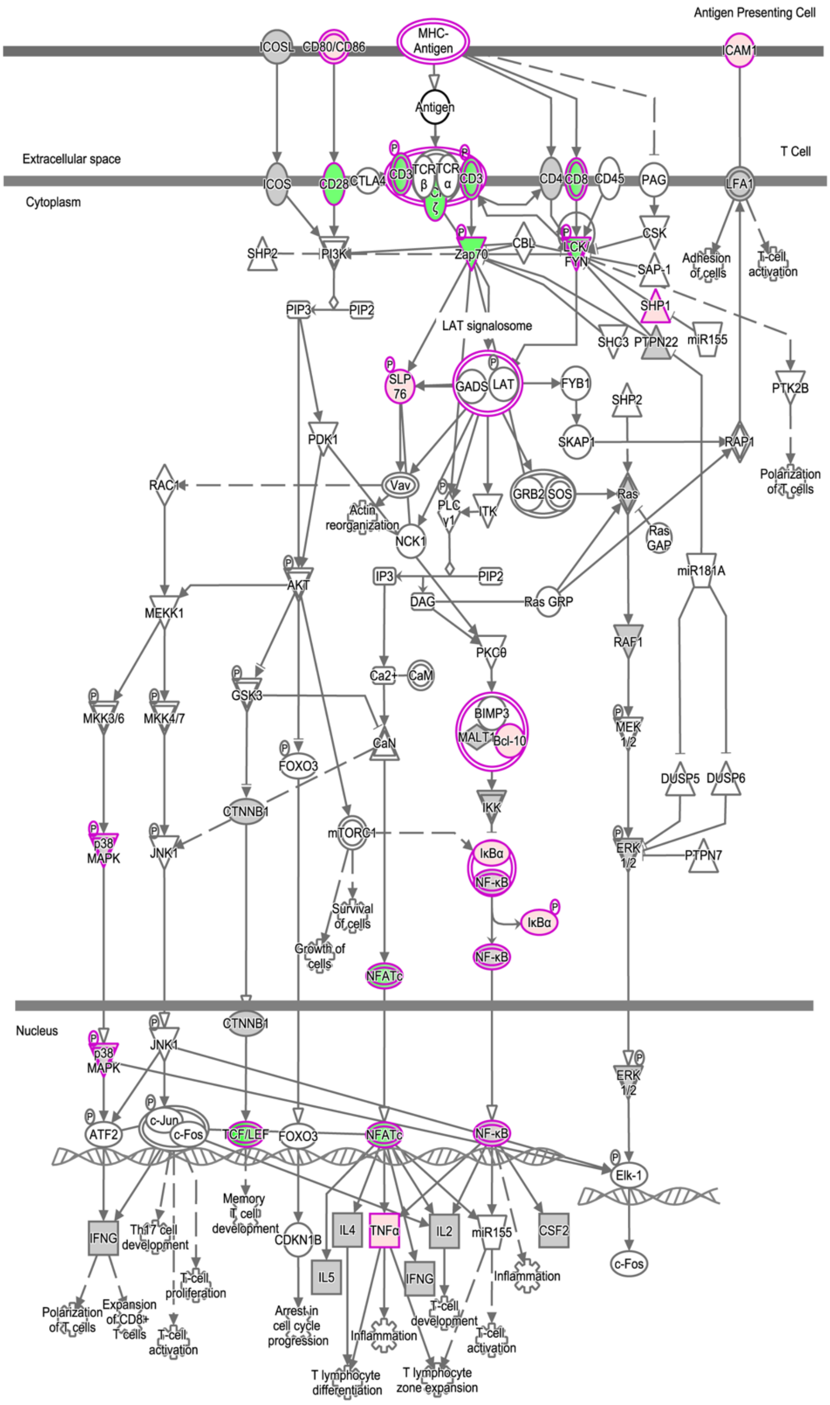

Supplementary figure 7. Transcripts related to T cell signalling are differentially expressed one day following vaccination. Ingenuity pathway analysis showing transcripts involved in T cell signalling that are significantly altered on day 2 (False Discovery Rate [FDR]-adjusted p-value < 0.05, Benjamini-Hochberg step-up procedure). Pink ovals indicate transcripts that are upregulated whereas green ovals indicate transcripts that are downregulated.

Supplementary Fig. 8

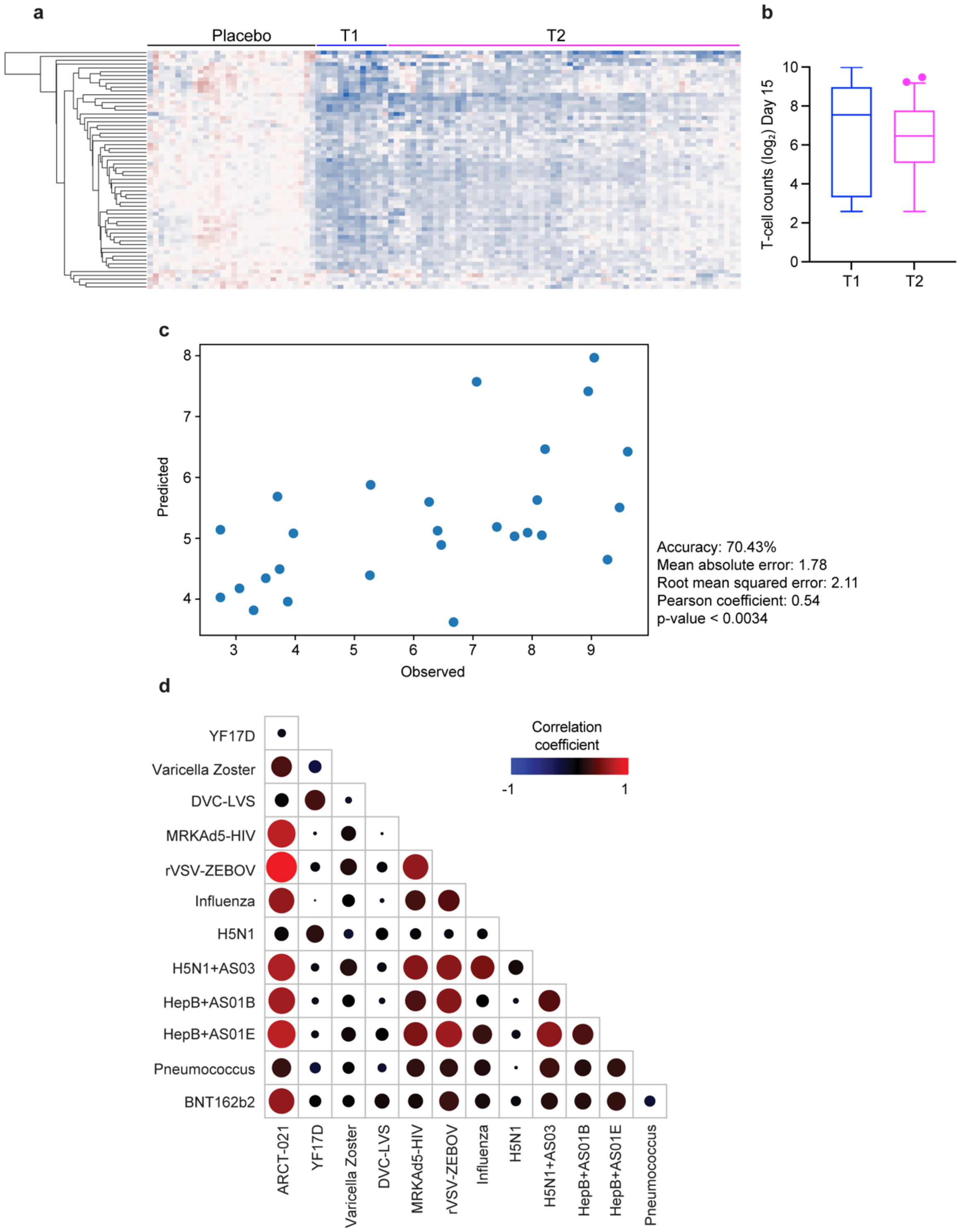

Supplementary figure 8. **Gene signatures at day 1 post-vaccination poorly predict T cell responses.** a. Gene and sample hierarchical clustering based on expression profiles of downregulated DEGs on day 2. Three distinct sample clusters, namely placebo, T1 and T2 are detected. Magnitude of downregulation is proportional to the colour intensity. **b.** Spike-specific T cell responses measured at day 15 in

subjects from T1 and T2 clusters. Box plots in **b** represent 25%-75% intervals, with lines indicating medians. The whiskers represent 10%-90% intervals. **c.** Scatterplot of the predicted and observed antibody titers, where predicted values were calculated based on the random forest regression model using log2-transformed fold change values of CD27, PSMB5 and LEF1 on day 8. Accuracy, mean absolute error, root mean-squared error, Pearson coefficient and p-values of the relationship between predicted and observed values are presented. **d.** Correlation matrix showing pairwise correlations of the mean gene log2-transformed fold changes between the different vaccines at day 1 post vaccination. Size and intensity of dots are proportional to the magnitude of correlation coefficient.

S/N	Reference	Vaccine	Pathogen	Adjuvant / Vector	Vaccine type	Sample size	Database
1	Hou J et al., J Immunol, 2019	YF-17D	Yellow Fever	Live vaccine strain	Live-attenuated	N = 21	GSE82152
2	Li S et al., Cell, 2017	Zostavax® (VSV)	Varicella zoster	Live vaccine strain	Live-attenuated	N = 33	GSE79396
3	Nakaya HI et al., Nat Immunol, 2012	FluMist® (LAIV)	Seasonal Influenza	Live vaccine strain	Live-attenuated	N = 28	GSE29619
4	Goll JB et al., Vaccines, 2020	DVC-LVS	Francisella Tularensis	Live vaccine strain	Live-attenuated	N = 10	GSE149809
5	Zak DE et al., PNAS, 2012	MRKAd5/HIV	HIV	Ad5	Recombinant viral vector	N = 7	GSE22822
6	Santoro F et al., Vaccines, 2021	rVSV-ZEBOV	Ebola	VSV	Recombinant viral vector	N = 51	Zenodo 3974486
7	Nakaya HI et al., Immunity, 2015	TIV	Seasonal Influenza	None	Inactivated	N = 19	GSE74813
8	Kazmin DA et al., Unpublished	H5N1	H5N1 Influenza	None	Inactivated	N = 16	GSE102012
9	Kazmin DA et al., Unpublished	H5N1 + AS03	H5N1 Influenza	AS03	Inactivated	N = 33	GSE102012
10	De Mot L et al., Sci Transl. Med., 2020	HepB + AS01B	Hepatitis B	AS01B	Inactivated	N = 18	GSE116975
11	De Mot L et al., Sci Transl. Med., 2020	HepB + AS01E	Hepatitis B	AS01E	Inactivated	N = 23	GSE116975
12	Obermoser G et al., Immunity, 2013	Pneumovax23	Pneumococcal bacteria	None	Polysaccharide	N = 6	GSE30101
13	Arunachalam PS et al., Nature, 2021	BNT162b2	SARS-CoV-2	mRNA-LNP	mRNA	N = 32	GSE169159

Supplementary Table 1: Vaccine studies used for comparative analysis with ARCT-021.
