# Supplementary material for: Immune gene expression analysis indicates the potential of a self-amplifying Covid-19 mRNA vaccine
Source: NPJ Vaccines. 2022 Nov 28;7:154. doi: 10.1038/s41541-022-00573-y (PMC9703414; doi:10.1038/s41541-022-00573-y)
Supplement: Supplementary file 2 — REPORTING SUMMARY [file 41541_2022_573_MOESM2_ESM.pdf]

nature portfolio

Corresponding author(s): Eng Eong Ooi, Kuan Rong Chan

Last updated by author(s): Oct 20, 2022

Reporting Summary

Nature Portfolio wishes to improve the reproducibility of the work that we publish. This form provides structure for consistency and transparency in reporting. For further information on Nature Portfolio policies, see our Editorial Policies and the Editorial Policy Checklist.

Statistics

For all statistical analyses, confirm that the following items are present in the figure legend, table legend, main text, or Methods section.

n/a	Confirmed	
☐	☒	The exact sample size (*n*) for each experimental group/condition, given as a discrete number and unit of measurement
☐	☒	A statement on whether measurements were taken from distinct samples or whether the same sample was measured repeatedly
☐	☒	The statistical test(s) used AND whether they are one- or two-sided<br>*Only common tests should be described solely by name; describe more complex techniques in the Methods section.*
☐	☒	A description of all covariates tested
☐	☒	A description of any assumptions or corrections, such as tests of normality and adjustment for multiple comparisons
☐	☒	A full description of the statistical parameters including central tendency (e.g. means) or other basic estimates (e.g. regression coefficient) AND variation (e.g. standard deviation) or associated estimates of uncertainty (e.g. confidence intervals)
☐	☒	For null hypothesis testing, the test statistic (e.g. *F*, *t*, *r*) with confidence intervals, effect sizes, degrees of freedom and *P* value noted<br>*Give P values as exact values whenever suitable.*
☒	☐	For Bayesian analysis, information on the choice of priors and Markov chain Monte Carlo settings
☒	☐	For hierarchical and complex designs, identification of the appropriate level for tests and full reporting of outcomes
☐	☒	Estimates of effect sizes (e.g. Cohen's *d*, Pearson's *r*), indicating how they were calculated

Our web collection on statistics for biologists contains articles on many of the points above.

Software and code

Policy information about availability of computer code

Data collection	Raw and normalised counts were derived with the NanoString nSolver 4.0 software.
Data analysis	Differentially expressed genes (DEGs) were defined as genes with fold-change > 1.3 and False Discovery Rate [FDR]-adjusted p-value < 0.05, Benjamini-Hochberg step-up procedure in Partek Genomics Suite v7.18. Ranking and identification of enriched pathways was based on adjusted p-values < 0.05, using GSEApy (https://github.com/ostrokach/gseapy). Unpaired, two-sided Student's t-test was used for comparisons between vaccinated groups using Prism v.9.1 software (GraphPad Software Inc.).

For manuscripts utilizing custom algorithms or software that are central to the research but not yet described in published literature, software must be made available to editors and reviewers. We strongly encourage code deposition in a community repository (e.g. GitHub). See the Nature Portfolio guidelines for submitting code & software for further information.

Data

Policy information about availability of data

All manuscripts must include a data availability statement. This statement should provide the following information, where applicable:
- Accession codes, unique identifiers, or web links for publicly available datasets
- A description of any restrictions on data availability
- For clinical datasets or third party data, please ensure that the statement adheres to our policy

The raw data and log2 fold change values for NanoString profiling of immune responses are available at Array Express (E-MTAB-11315).

Human research participants

Policy information about studies involving human research participants and Sex and Gender in Research.

Reporting on sex and gender	A total of 78 male healthy volunteers and 28 female healthy volunteers were recruited for this study. Information on sex of volunteers was collected as part of demographic information following written informed consent. Vaccine immunogenicity of ARCT-021 was not found to be influenced by sex, hence sex-based analysis was not performed in this study.
Population characteristics	All subjects recruited were healthy adults, aged 21 to 55 years old (younger adult cohorts) or 56 to 80 years old (older adult cohorts) at the time of informed consent. For more details on the population characteristics for the study, please refer to Low et al., medRxiv, 2021:2021.07.01.21259831, Table 1.
Recruitment	Healthy volunteers were recruited from the SingHealth Investigational Medicine Unit healthy volunteer database, and IRB-approved advertisements.
Ethics oversight	SingHealth Centralized Institutional Review Board (CIRB/F 2020/2553) and the Singapore Health Sciences Authority

Note that full information on the approval of the study protocol must also be provided in the manuscript.

Field-specific reporting

Please select the one below that is the best fit for your research. If you are not sure, read the appropriate sections before making your selection.

☒ Life sciences ☐ Behavioural & social sciences ☐ Ecological, evolutionary & environmental sciences

For a reference copy of the document with all sections, see nature.com/documents/nr-reporting-summary-flat.pdf

Life sciences study design

All studies must disclose on these points even when the disclosure is negative.

Sample size	No formal sample size calculation was performed. Based on experience from previous studies with other RNA based therapies, the chosen cohort sizes were considered sufficient to meet the objectives of the study while minimizing unnecessary subject exposure.
Data exclusions	No data was excluded.
Replication	nCounter probe sets include negative and positive controls for each sample lane that are used for background thresholding and data normalization. There were no flags for positive control linearity quality control (QC) or limit of detection QC in nSolver.<br>Enriched pathways were ranked and identified based on adjusted p-values < 0.05, by running GSEApy with 1000 permutations to generate enrichment statistics.
Randomization	The Sponsor or designee prepared the randomization list, which was provided to the study site unblinded pharmacist.
Blinding	Investigators were blinded to group allocation.

Reporting for specific materials, systems and methods

We require information from authors about some types of materials, experimental systems and methods used in many studies. Here, indicate whether each material, system or method listed is relevant to your study. If you are not sure if a list item applies to your research, read the appropriate section before selecting a response.

Materials & experimental systems

n/a	Involved in the study	
☒	☐	Antibodies
☒	☐	Eukaryotic cell lines
☒	☐	Palaeontology and archaeology
☒	☐	Animals and other organisms
☐	☒	Clinical data
☒	☐	Dual use research of concern

Methods

n/a	Involved in the study	
☒	☐	ChIP-seq
☒	☐	Flow cytometry
☒	☐	MRI-based neuroimaging

Clinical data

Policy information about clinical studies

All manuscripts should comply with the ICMJE guidelines for publication of clinical research and a completed CONSORT checklist must be included with all submissions.

Clinical trial registration	ClinicalTrials.gov NCT04480957
Study protocol	ClinicalTrials.gov NCT04480957
Data collection	The study was conducted at the SingHealth Investigation Medicine Unit in Singapore. Study start date was Aug 4 2020 and study completion date was 29 Jan 2021.
Outcomes	Nanostring profiling of host response for key immune regulatory genes was performed using the nCounter Human Immunology v2 Panel at day 1 (pre-dose 1), 2, 3, and 8 for all vaccinees. Gene expression was profiled at day 29 (pre-dose 2), 30 and 36 among vaccinees in the two-injection cohort.
